# Impact of the COVID-19 Pandemic on Non-COVID-19 Clinical Trials

**DOI:** 10.3390/jcdd9010019

**Published:** 2022-01-10

**Authors:** Katia Audisio, Hillary Lia, Newell Bryce Robinson, Mohamed Rahouma, Giovanni Soletti, Gianmarco Cancelli, Roberto Perezgrovas Olaria, David Chadow, Derrick Y. Tam, Dominique Vervoort, Michael E. Farkouh, Deepak L. Bhatt, Stephen E. Fremes, Mario Gaudino

**Affiliations:** 1 Department of Cardiothoracic Surgery, Weill Cornell Medicine, New York, NY 10065, USA; kaa4008@med.cornell.edu (K.A.); ner7006@nyp.org (N.B.R.); mmr2011@med.cornell.edu (M.R.); gis2011@med.cornell.edu (G.S.J.); gic4005@med.cornell.edu (G.C.); rop4006@med.cornell.edu (R.P.O.); dac4015@med.cornell.edu (D.C.); 2 Schulich Heart Centre, Sunnybrook Health Sciences Centre, University of Toronto, Toronto, ON M4N 3M5, Canada; hillary.lia@mail.utoronto.ca (H.L.); derricky.tam@gmail.com (D.Y.T.); d.vervoort@mail.utoronto.ca (D.V.); Stephen.fremes@sunnybrook.ca (S.E.F.); 3 Peter Munk Cardiac Centre, University of Toronto, Toronto, ON M5G 2N2, Canada; michael.farkouh@uhn.ca; 4 Division of Cardiovascular Medicine, Brigham and Women’s Hospital, Harvard Medical School, Boston, MA 02115, USA; DLBhattMD@post.Harvard.Edu

**Keywords:** COVID-19, randomized controlled trials, ClinicalTrials.gov

## Abstract

Randomized controlled trials (RCT) were impacted by the COVID-19 pandemic, but no systematic analysis has evaluated the overall impact of COVID-19 on non-COVID-19-related RCTs. The ClinicalTrials.gov database was queried in February 2020. Eligible studies included all randomized trials with a start date after 1 January 2010 and were active during the period from 1 January 2015 to 31 December 2020. The effect of the pandemic period on non-COVID-19 trials was determined by piece-wise regression models using 11 March 2020 as the start of the pandemic and by time series analysis (models fitted using 2015–2018 data and forecasted for 2019–2020). The study endpoints were early trial stoppage, normal trial completion, and trial activation. There were 161,377 non-COVID-19 trials analyzed. The number of active trials increased annually through 2019 but decreased in 2020. According to the piece-wise regression models, trial completion was not affected by the pandemic (*p* = 0.56) whereas trial stoppage increased (*p* = 0.001). There was a pronounced decrease in trial activation early during the pandemic (*p* < 0.001) which then recovered. The findings from the time series models were consistent comparing forecasted and observed results (trial completion *p* = 0.22; trial stoppage *p* < 0.01; trial activation, *p* = 0.01). During the pandemic, there was an increase in non-COVID-19 RCTs stoppage without changes in RCT completion. There was a sharp decline in new RCTs at the beginning of the pandemic, which later recovered.

## 1. Introduction

The Coronavirus Disease 2019 (COVID-19) pandemic has had a profound impact on all areas of the healthcare system, including clinical research [1]. Research funding was diverted to the initiation of trials related to addressing the COVID-19 pandemic and development of vaccines, researchers were restricted from accessing laboratory space, and trial participants were unable to attend routine follow-up visits for non-COVID-19 research-related care [2].

Randomized controlled trials (RCT) represent the gold standard for biomedical research [3]. Faced with the challenges of the COVID-19 pandemic, RCTs had to adjust protocols to accommodate the sudden suspension of recruitment, in-person data collection, and safety visits, as well as delivery of interventions, which may lead to a pause or premature closure of the trial.

Prior analyses have attempted to characterize the impact of the pandemic on trial status, funding, and recruitment. These analyses, however, were published early in the pandemic [4] or focused on COVID-19-related RCTs [5]. To date, no systematic analysis has evaluated the overall impact of COVID-19 on non-COVID-19-related RCTs. In this analysis, we characterize the effect of the COVID-19 pandemic on non-COVID-19 RCTs.

## 2. Materials and Methods

### 2.1. Data Source

The ClinicalTrials.gov database was queried using Python version 3.9 (Python Software Foundation, Beaverton, OR, USA) on 11 February 2020. The ClinicalTrial.gov database has been previously described [6]. The database is the largest registry of RCTs in the world, with trials from over 180 countries. All trials containing keywords “COVID-19”, “SARS-CoV-2”, and “coronavirus” were excluded from the initial import of the data. Records were obtained and loaded into a Pandas dataframe [7] for further processing. Trial fields obtained were “NCTId”, “Brief Title”, “Lead Sponsor Class”, “Lead Sponsor Name”, “Location Country”, “Registration Date,” “Start Date”, “Phase, “Completion Date”, “Last Update Post Date”, “Intervention Type”, “Study Type”, “Healthy Volunteers”, “Standardized Age” and “Last Known Status”. A full list of trial field definitions is available in Table S1.

Trials were considered “Completed” if the trial was completed within the expected timeline and “Stopped” if their last known status on ClinicalTrials.gov was either “Suspended”, “Terminated”, or “Withdrawn”. The stop date of a trial was obtained from the “Last Update Post Date” datafield.

Trials which were registered without a start date were not included. Any study listed as “Observational” was also excluded. In order to understand the usual contemporary pattern for trial initiation, stoppage, and completion prior to the pandemic, we included all trials with a start date after 1 January 2010 and which were active during the period from 1 January 2015 to 31 December 2020. Trials which were completed or stopped prior to 1 January 2015 and those with a start date prior to 1 January 2010 or later than 31 December 2020 were excluded. The pandemic period was defined as 11 March 2020 (the date that the World Health Organization declared the COVID-19 pandemic) through 31 December 2020.

Columns for each calendar year and the pandemic period itself were created in the dataframe to indicate whether a trial was “Active”, “Completed”, or “Stopped” during each time period. A trial was “Active” within any calendar year if its “Start Date” was within or prior to the same calendar year and the trial was not “Completed” or “Stopped” prior to the end of the same year. A trial was “Completed” or “Stopped” if the completion or stop date was within the same calendar year.

### 2.2. Study Cohorts

Trials which were completed or stopped prior to 11 March 2020 were included in the pre-pandemic cohort (PRE). Trials which were active during the pandemic, regardless of the start date, were included in the pandemic cohort (PAN).

### 2.3. Study Outcomes

The primary study outcomes were trial completion and trial stoppage. Trial initiation was the secondary outcome.

### 2.4. Statistical Analysis

Categorical variables were reported as frequency counts and percentages and compared using X^2^ and Fisher’s exact tests as appropriate. Continuous variables were expressed as median and inter-quartile range and compared using a Mann–Whitney test after assessment of normality. For the calculation of the percentage of studies stopped or completed within a certain year or month, the denominator was defined as the number of trials active at the beginning of the year or month plus the numbers of trials initiated within that year or month without further correction. For the calculation of the percentage of trials initiated, the denominator was defined as the numbers of trials open at the beginning of that year or month. For monthly calculations, March 2020 was considered as a pandemic month. The trends over time in terms of the absolute numbers of trials stopped, completed, or activated are depicted graphically.

In addition, the effects of the pandemic on non-COVID-19 trials were assessed using piece-wise regression and using time series ARIMA models. Piece-wise regression models were used to estimate the difference in the numbers of stopped, completed, or activated trials over time, using 11 March 2020 as the start of the pandemic. An additional variable including the months since 11 March 2020 was created to determine if the pandemic effect was sustained or transitory.

Time series models were fit for each endpoint using monthly values from 2015–2018 with the Auto ARIMA function through the Python pmdarima package. The Augmented Dickey–Fuller test was applied to each to assess for stationarity of the data. Non-stationarity was accounted for by the Auto ARIMA function. Monthly values for 2019 and 2020 were forecasted using the fitted model and plotted with the actual values to assess differences over time. Confidence intervals at 95% were plotted for forecasted values. Independence for model residuals was evaluated using autocorrelation to white noise assessment. The forecasted curve was smoothed using Holt’s exponential smoothing from the Statsmodels package. Parameters were specified as a smoothing level of 0.6 and a smoothing trend of 0.2 for stopped trials and were selected by the Holt function for completed and initiated trials. The smoothed curve was plotted alongside the real data and forecasted curves.

Two-sided significance testing was used and a *p*-value < 0.05 was considered significant without adjustment for multiple testing. Statistical analyses were performed using R (version 3.4.2 R Project for Statistical Computing) within RStudio.

## 3. Results

The details of the study cohort are depicted in Figure S1. The initial query of the ClinicalTrials.gov database yielded 361,899 results. After excluding trials with a start date earlier than 1 January 2010, trials with start dates after our study period, and trials which were stopped or completed prior to our study period, a total of 212,286 records remained. We included 161,377 non-COVID-19 trials in the final analysis after removal of 50,909 observational studies and 2043 records with incomplete data. Over the study period, 121,409 trials were initiated, 65,685 were completed and 11,964 were stopped. Table S2 and Figures S2 and S3 depict the annual changes over time of trial status. Overall, there was an increasing number of non-COVID-19 trials initiated from 1 January 2015 through to 2019, with a reduction in 2020.

The characteristics of trials overall and separately for the PRE and PAN cohorts are summarized in Table 1.

Industry was the sponsor of 35,669 trials (22.1%) and a federal agency was the sponsor of 8376 (5.2%) trials. About one-third (49,421 [30.6%]) of the trials were Phase 1 or 2, and 26,364 (16.3%) were Phase 3 or 4. Device interventions were used in 19,212 (11.9%) trials, and drugs in 53,618 (33.2%) trials. A majority (134,532 [83.4%]) of trials enrolled adult patients and only 42,301 (26.2%) included healthy volunteers. Trials originated most commonly from North America, Europe, and Asia (59,701 [37.0%], 34,409 [21.3%], 30,894 [19.1%], respectively).

### Trends over Time and Pandemic Effect

**Figure 1:**
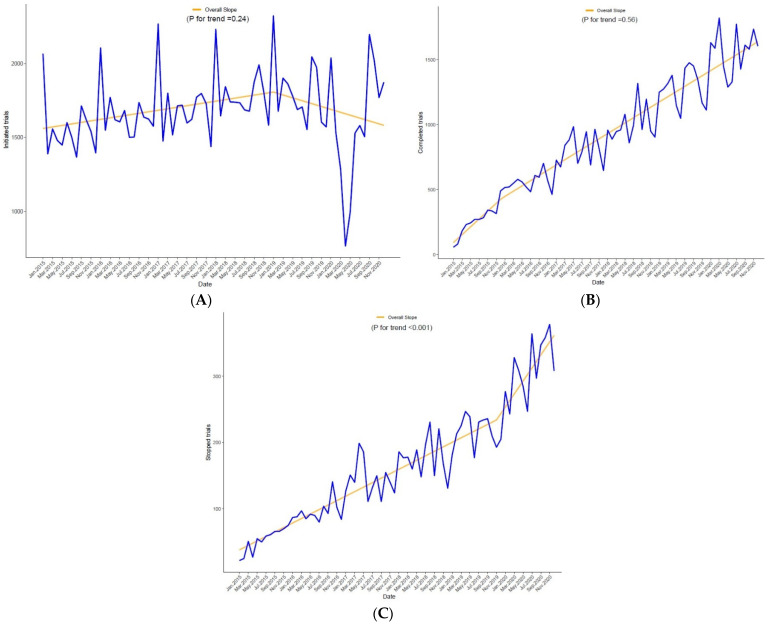
Time segmented analysis for (**A**) stopped trials, (**B**) completed trials, and (**C**) initiated trials. *p*-values were derived using multivariable piecewise linear regression.

**Figure 2:**
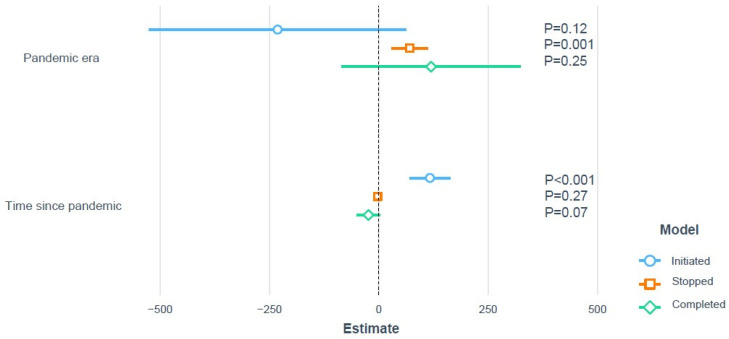
Piecewise linear regression of monthly trend of initiated, stopped and completed trials during the pandemic era and time since pandemic based on monthly number and percentages, respectively.

There was a statistically significant increase in the number of trials stopped during the pandemic (*p* = 0.001), whereas there was no effect of the pandemic on trial completion (*p* = 0.56) (Figure 1A–C and Figure S4A–C). There was a sharp reduction in trial initiation in the COVID-19 period, which later recovered, (Figure 1C and Figure 2).

**Figure 3:**
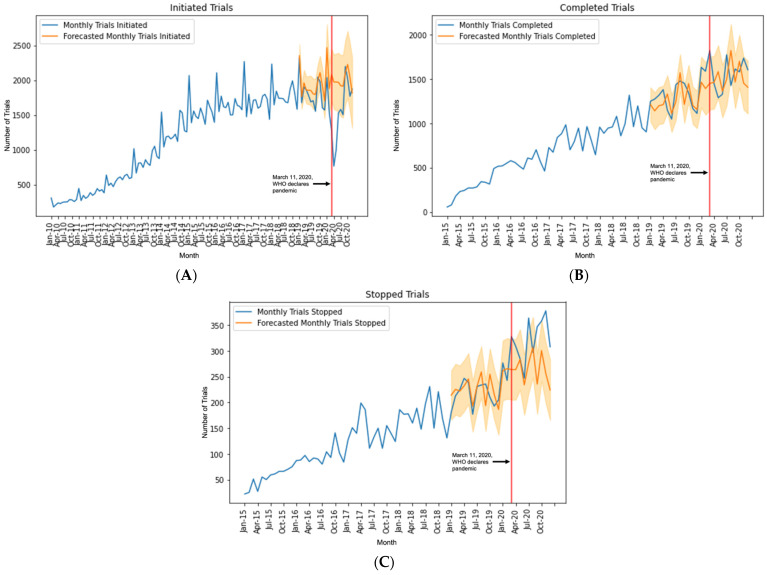
Trends in monthly trial (**A**) initiation, (**B**) completion, and (**C**) stoppage before and after declaration of the COVID-19 pandemic. Actual number of trials (blue line) and predicted trials (orange line). The orange shading represents the forecast’s 95% confidence intervals.

In the SARIMA model that examined the number of new monthly initiated trials, there was a decrease in new trial initiations in the months of March, April, May, and June 2020 beyond 95% CI bounds (Figure 3A).

The largest decrease was in April 2020 (−61.3%; actual: 765, forecasted: 1976, 95% CI: 1585–2368) (Table 2).

The number of monthly completed trials was above the 95% CI bounds of the forecasted model for the month of March 2020 only (+25.6%; actual: 1822, forecasted: 1451, 95% CI: 1153–1748) (Figure 3B and Table 2).

In the fitted SARIMA model, the actual number of monthly stopped trials for the months of March, July, September, November, and December 2020 exceeded the forecasted values of the model beyond 95% CI bounds (Figure 3C). The greatest increase occurred in November 2020 (+47.7%; actual: 378, forecasted: 256, 95% CI: 198–315) (Table 2). Tables S3–S5 display SARIMA model fit and summary statistics.

## 4. Discussion

In this analysis, we found that during the COVID-19 pandemic, there was a significant increase in the stoppage of non-COVID-19-related trials compared to the non-pandemic years, with no difference in trial completion. Furthermore, there was a marked decrease in non-COVID-19 trial initiation early in the pandemic, which rebounded later during 2020.

We believe that the differential effect of the pandemic on trial completion and stoppage is related to the fact that trials initiated before the pandemic and therefore at a further stage were more likely to be completed during the pandemic, whereas trials started during the pandemic or at an earlier stage were stopped at a higher rate. Indeed, the distribution of the start years was heavily weighted towards the earlier years in the pre-pandemic period and U-shaped in the pandemic period.

Prior reports showed a significant reduction in research activities during the current pandemic. In an international survey of 60 large academic cardiac surgery centers, research activity was reported to be reduced by more than 50% from pre-pandemic levels [8]. A report on oncology trials found that up to 20% of centers in Asia and the US had stopped enrollment entirely between March and April 2020, with 60% of centers reporting continued enrollment but at a lower rate when compared with the pre-pandemic era [9]. In a survey of 933 oncology patients, Fleury et al. found that 20% of respondents reported that they were less likely to participate in clinical research, citing the ongoing COVID-19 pandemic and restricted access to care [10]. The US National Institute of Health estimated that almost 80% of non-COVID-19 clinical trials were stopped or interrupted as a result of the pandemic [11]. Even for trials that were not stopped, enrollment was problematic during the COVID-19 crisis. Sathian et al., in a systematic review evaluating the impact of the COVID-19 pandemic on the conduction of clinical trials, found that most sites conducting clinical trials not related to COVID-19 experienced a delay in timelines or a complete stoppage during the pandemic, [12]. Selvaraj et al., in an analysis on the topics of cardiovascular trials before and during the COVID-19 pandemic, found that most of the new trials focused exclusively on COVID-19-retaled cardiovascular outcomes. [13] These analyses, however, were published early in the pandemic, focused only on specific clinical subspecialties, or did not specifically select non-COVID-19 trials. The novelty of our analysis is that we performed the first systematic analysis evaluating the overall impact of the COVID-19 on non-COVID-19 RCTs and their initiation, stoppage, and completion.

Clinical trials may provide patients with access to experimental therapies and new medications; when suspended or prematurely stopped, patients lose this crucial resource. Oncology trials, for example, often enroll patients who have few, if any, alternative treatment options by offering personalized treatment modalities such as immunotherapy [14].

Initiation of new trials not related to COVID-19 therapies was similarly impacted. In an analysis of Phase 1 through 4 oncology trials, Lamont et al. found that when compared with the pre-pandemic period, the COVID-19 pandemic was associated with a 60% decrease in the initiation of new trials [15]. Although the exact long-term consequences remain unknown, the decrease in trial initiation will likely delay the development of new therapies for the foreseeable future.

With the sustained strain on hospitals caused by the COVID-19 pandemic, the modification and suspension of clinical research is likely to continue. At the start of the pandemic, many academic medical centers suspended all in-person research activities whereas others left the decision up to the discretion of the primary investigator [16]. Although it is clear that essential research activities need to continue, its definition remains elusive [17]. Continued restriction in patient movement and hospital activities will impact data collection and monitoring visits, compliance with the investigated therapy, and other protocol-related requirements.

In response to the COVID-19 crisis, the United States Food and Drug Administration (FDA) and European Medicine Agency (EMA) issued guidance for industry, investigators, and institutional review boards [18,19]. Recommendations include virtual or in-home study visits, shipment of interventional medications to the patients’ home, and other forms of remote monitoring. Although these are important steps in the right direction, these temporizing measures do not fully address the future of clinical research in a post-COVID-19 environment.

Trial design needs to adapt to this changing landscape to allow for the continuation of crucial, non-COVID-19-related research, as well as prepare for possible future disruptions. Conventional clinical trials are typically run from clinical sites where patients report for face-to-face interaction with study personnel and undergo study-related treatment and testing. This structure is fragile in the case of pandemic crises similar to COVID-19, as it carries the risk of exposure to infection of study participants [20]. By allowing virtual and at-home consenting and subsequent care, trials may be able to recruit potential participants even during a crisis similar to the current pandemic. This would require the restructuring of protocols such as that patients could perform the functions of research personnel with little to no assistance [21]. Digital devices can also collect symptom scores and baseline vital data that can be electronically transmitted to central data repositories [22]. During the COVID-19 pandemic, a number of trials have successfully been conducted without a physical clinical site [23,24]. A restructuring of trials without clinical sites offers several advantages such as increased access to treatments for patients located further from clinical sites, decreased cost, and a more patient-centered approach [25].

Our analysis should be interpreted in the context of several limitations. First, the ClinicalTrials.gov database is constantly updated, and these results reflect trial status as of February 2021. Second, although the ClinicalTrials.gov database is the largest clinical trial registry in the world, there is a potential for missing unregistered trials, and we did not search for trials registered to other registries. Third, the ClinicalTrials.gov database relies on compliance by individual study investigators to update their status, and there is a potential that some studies may have been suspended or stopped without updating their status on the database; it is also possible that there have been errors in data entry and trial status reporting. The rates of trial stoppage, either temporary or permanent, may be underreported. Furthermore, the design type of the clinical trials and the subject matter breakdown were not available in the ClinicalTrials.gov database; therefore, a sub-analysis evaluating if the trials with these variables were affected by the COVID-19 pandemic was not possible.

## 5. Conclusions

To conclude, the COVID-19 pandemic has had a significant impact on non-COVID-19-related clinical trials. There has been a significantly higher number of stopped trials in addition to a significantly lower number of newly initiated trials. Trial design and protocols have to rapidly change and adapt to the post-COVID-19 world. Virtual visits, digital monitoring, and remote delivery of study interventions can allow not only for “future-proofing” of clinical trials, but also allow for increased participation with decreased cost.

## Figures and Tables

**Table 1:** Characteristics of the trials in the pre-pandemic (PRE) and pandemic (PAN) era.

Variable	Total (*n* = 161,377)	PRE ^a^ (*n* = 59,478)	PAN (*n* = 101,899)	*p*-Value	SMD
Sponsor Class	-	-	-	<0.001	0.17
• Federal	8376 (5.2)	2658 (4.5)	5718 (5.6)	-	-
• Individual	162 (0.1)	83 (0.1)	79 (0.1)	-	-
• Industry	35,669 (22.1)	15,797 (26.6)	19,872 (19.5)	-	-
• Other	117,170 (72.6)	40,940 (68.8)	76,230 (74.8)	-	-
Study Phase ^b^	-	-	-	<0.001	0.02
• Phase 1 and/or 2	49,421 (30.6)	17,917 (30.1)	31,504 (30.9)	-	-
• Phase 3 and/or 4	26,364 (16.3)	9982 (16.8)	16,382 (16.1)	-	-
• Not Applicable	85,592 (53.0)	31,579 (53.1)	54,013 (53.0)	-	-
Intervention Type	-	-	-	<0.001	0.14
• Device	19,212 (11.9)	7012 (11.8)	12,200 (12.0)	-	-
• Diagnostic	1976 (1.2)	350 (0.6)	1626 (1.6)	-	-
• Drug	53,618 (33.2)	20,682 (34.8)	32,936 (32.3)	-	-
• Other	74,082 (45.9)	27,619 (46.4)	46,463 (45.6)	-	-
• Procedure	11,108 (6.9)	3530 (5.9)	7578 (7.4)	-	-
• Radiation	1381 (0.9)	285 (0.5)	1096 (1.1)	-	-
Healthy Volunteers	-	-	-	<0.001	0.28
• Accepted	42,301 (26.2)	18,589 (31.3)	23,712 (23.3)	-	-
• Not Accepted	119,076 (73.8)	40,889 (68.7)	78,187 (76.7)	-	-
Age group included	-	-	-	<0.001	0.04
• Adults and Older Adults	134,532 (83.4)	49,742 (83.6)	84,790 (83.2)	-	-
• Children Only	10,029 (6.2)	3913 (6.6)	6116 (6.0)	-	-
• Children, Adults, and Older Adults	16,816 (10.4)	5823 (9.8)	10,993 (10.8)	-	-
Continent	-	--	-	<0.001	0.17
• Africa	5031 (3.1)	1731 (2.9)	3300 (3.2)	-	-
• Antarctica	1 (0.0)	0 (0.0)	1 (0.0)	-	-
• Asia	30,894 (19.1)	9325 (15.7)	21,569 (21.2)	-	-
• Europe	34,409 (21.3)	13,619 (22.9)	20,790 (20.4)	-	-
• Multiple	12,373 (7.7)	4133 (6.9)	8240 (8.1)	-	-
• North America	59,701 (37.0)	22,812 (38.4)	36,889 (36.2)	-	-
• Oceania	871 (0.5)	332 (0.6)	539 (0.5)	-	-
• South America	3306 (2.0)	1319 (2.2)	1987 (1.9)	-	-
• Unknown	14,791 (9.2)	6207 (10.4)	8584 (8.4)	-	-
Start Year	-	-	-	<0.001	1.19
• Before 2015	39,968 (24.8)	25,095 (42.2)	14,873 (14.6)	-	-
• 2015	18,684 (11.6)	10,835 (18.2)	7849 (7.7)	-	-
• 2016	19,917 (12.3)	9514 (16.0)	10,403 (10.2)	-	-
• 2017	20,453 (12.7)	7427 (12.5)	13,026 (12.8)	-	-
• 2018	21,561 (13.4)	4802 (8.1)	16,759 (16.4)	-	-
• 2019	21,693 (13.4)	1776 (3.0)	19,917 (19.5)	-	-
• 2020	19,101 (11.8)	29 (0.0)	19,072 (18.7)	-	-

Data presented as *n* (%), unless otherwise noted. ^a^ Pre-pandemic includes all active trials prior to 11 March 2020. ^b^ Trials classified as Phase 2/3 were included in the Phase 1 and/or 2. SMD: standardized mean difference, PAN: pandemic era, PRE: pre-pandemic era.

**Table 2:** Number of observed and predicted initiated, completed, and stopped non-COVID-19 trials between March and December 2020.

Month	Observed Trials (*n*°)	Forecasted Trials (*n*°)	95% Confidence Interval	Difference (%)
**Initiated Trials**				
March 2020	1285	2079	1704–2454	−38.2%
April 2020	765	1976	1585–2368	−61.3%
May 2020	996	1974	1566–2382	−49.5%
June 2020	1530	1970	1547–2394	−22.3%
July 2020	1581	1922	1484–2361	−17.7%
August 2020	1505	1913	1460–2366	−21.3%
September 2020	2198	2111	1644–2578	+4.1%
October 2020	2022	2227	1747–2708	−9.2%
November 2020	1770	2042	1549–2536	−13.3%
December 2020	1874	1818	1312–2325	+3.1%
**Completed Trials**				
March 2020	1822	1451	1153–1749	+25.6%
April 2020	1449	1462	1164–1761	−0.9%
May 2020	1289	1583	1285–1882	−18.6%
June 2020	1329	1362	1064–1661	−2.4%
July 2020	1774	1498	1200–1797	+18.4%
August 2020	1427	1821	1523–2120	−21.6%
September 2020	1613	1465	1167–1764	−10.1%
October 2020	1581	1700	1402–1999	−7.1%
November 2020	1736	1453	1155–1752	+19.5%
December 2020	1604	1407	1109–1706	+14%
**Stopped Trials**				
March 2020	328	264	205–323	+24.2
April 2020	309	264	205–323	+17.0
May 2020	285	283	224–342	+0.7
June 2020	247	234	176–293	+5.6
July 2020	364	277	218–336	+31.4
August 2020	297	307	248–366	−3.3
September 2020	347	236	177–295	+47.0
October 2020	358	301	242–359	+18.9
November 2020	378	256	198–315	+47.7
December 2020	308	224	166–283	+37.5

## Data Availability

Data collected for the study will be made available by the corresponding author upon reasonable request after publication.

## Supplementary Materials

The following are available online https://www.mdpi.com/article/10.3390/jcdd9010019/s1. Table S1: Full list of trial definitions, Table S2: Annual changes over time in trial status, Table S3: SARIMA model fit and summary statistics for stropped trials, Table S4: SARIMA model fit and summary statistics for initiated trials, Table S5: SARIMA model fit and summary statistics for completed trials. Figure S1: Derivation of the Study Database, Figure S2: Yearly number of trials initiated (blue bar), stopped (orange bar), or completed (gray bar), Figure S3: Total number of trials that were active per year, Figure S4: (A) Total number of trials that were stopped over the study period by month, (B) Total number of trials that were completed over the study period by month, (C) Total number of trials that were initiated over the study period by month.
