# Supplementary material for: Impact of the COVID-19 Pandemic on Non-COVID-19 Clinical Trials
Source: J Cardiovasc Dev Dis. 2022 Jan 10;9(1):19. doi: 10.3390/jcdd9010019 (PMC8781416; doi:10.3390/jcdd9010019)

SUPPLEMENTAL MATERIAL

Supplemental Table S1. Full list of trial definitions [*]

Variable Name	Definition
**Start Date**	The actual date on which the first participant was enrolled in a clinical study. The "estimated" study start date is the date that the researchers think will be the study start date.
**Last Update Post Date**	The most recent date on which changes to a study record were made available on ClinicalTrials.gov.
**Status**	-
• **Suspended**	The study has stopped early but may start again.
• **Withdrawn**	The study stopped early, before enrolling its first participant.
• **Terminated**	The study has stopped early and will not start again. Participants are no longer being examined or treated.
• **Completed**	The study has ended normally, and participants are no longer being examined or treated (that is, the last participant's last visit has occurred).
**Location Country**	Countries in which research facilities for a study are located. A country is listed only once, even if there is more than one facility in the country.
**Sponsor Class**	The organization or person who initiates the study and who has authority and control over the study.
• **Individual**	Funded by individuals
• **Federal**	Other U.S. Federal agencies (for example, Food and Drug Administration, Centers for Disease Control and Prevention, or U.S. Department of Veterans Affairs)
• **Industry**	Industry (for example: pharmaceutical and device companies)
• **Others**	All others (including individuals, universities, and community-based organizations)
**Phase**	The stage of a clinical trial studying a drug or biological product, based on definitions developed by the U.S. Food and Drug Administration (FDA). The phase is based on the study's objective, the number of participants, and other characteristics. There are five phases: Early Phase 1 (formerly listed as Phase 0), Phase 1, Phase 2, Phase 3, and Phase 4. Not Applicable is used to describe trials without FDA-defined phases, including trials of devices or behavioral interventions.
• **Phase 1**	A phase of research to describe clinical trials that focus on the safety of a drug. They are usually conducted with healthy volunteers, and the goal is to determine the drug's most frequent and serious adverse events and, often, how the drug is broken down and excreted by the body. These trials usually involve a small number of participants.
• **Phase 2**	A phase of research to describe clinical trials that gather preliminary data on whether a drug works in people who have a certain condition/disease (that is, the drug's effectiveness). For example, participants receiving the drug may be compared to similar participants receiving a different treatment, usually an inactive substance (called a placebo) or a different drug. Safety continues to be evaluated, and short-term adverse events are studied.
• **Phase 3**	A phase of research to describe clinical trials that gather more information about a drug's safety and effectiveness by studying different populations and different dosages and by using the drug in combination with other drugs. These studies typically involve more participants.
• **Phase 4**	A phase of research to describe clinical trials occurring after FDA has approved a drug for marketing. They include post-market requirement and commitment studies that are required of or agreed to by the study sponsor. These trials gather additional information about a drug's safety, efficacy, or optimal use.
• **Not Applicable**	Describes trials without FDA-defined phases, including trials of devices or behavioral interventions.
**Intervention**	A process or action that is the focus of a clinical study. Interventions include drugs, medical devices, procedures, vaccines, and other products that are either investigational or already available. Interventions can also include noninvasive approaches, such as education or modifying diet and exercise.

**Healthy Volunteers**	A type of eligibility criteria that indicates whether people who do not have the condition/disease being studied can participate in that clinical study.
**Age Group**	A type of eligibility criteria that indicates the age a person must be to participate in a clinical study.
• **Child**	Birth to 17 years
• **Adult**	18 years to 64 years
• **Older Adult**	65+ years

* Definitions taken from ClinicalTrials.gov glossary.

Supplemental Table S2. Annual changes over time in trial status

Year	Trials Opened	Trials Completed	Trials Stopped
• **2015**	18,684	3,096	627
• **2016**	19,917	6,661	1,143
• **2017**	20,453	9,661	1,726
• **2018**	21,561	12,017	2,136
• **2019**	21,693	15,406	2,591
• **2020**	19,101	18,844	3,741
**Totals**	**121,409**	**65,685**	**11,964**
• **PRE**	34,383	50,639	8,839
• **PAN**	87,026	15,046	3,125

Supplemental Table S3. SARIMA model fit and summary statistics for stropped trials

SARIMA Summary Statistics (Stopped Trials)

**Number of Observations**	48					
**AIC**	342					
**BIC**	348					
	**Coefficient**	**Standard Error**	**Z**	**P>|z|**	**[0.025**	**0.975]**
**Intercept**	58.0498	12.433	4.669	0.000	33.681	82.418
**ma.L1**	0.2660	0.182	1.460	0.144	-0.091	0.623
**ar.S.L12**	-0.3650	0.165	-2.216	0.027	-0.688	-0.042
**sigma2**	598.5893	128.757	4.649	0.000	346.230	850.949

**Ljung-Box (L1) (Q)**	0.00	**Jarque-Bera (JB)**	5.07
**Prob (Q)**	0.97	**Prob(JB)**	0.08
**Heteroskedasticity (H)**	2.10	**Skew**	0.64
**Prob (H) (two-sided)**	0.21	**Kurtosis**	4.32

Supplemental Table S4. SARIMA model fit and summary statistics for initiated trials

SARIMA Summary Statistics (Initiated Trials)

**Number of Observations**	108					
**AIC**	1120					
**BIC**	1126					
	**Coefficient**	**Standard Error**	**Z**	**P>|z|**	**[0.025**	**0.975]**
**ma.L1**	-0.6629	0.075	-8.82-	0.000	-0.810	-0.516
**sigma2**	7392.4298	1004.689	7.358	0.000	5423.276	9361.584

**Ljung-Box (L1) (Q)**	0.09	**Jarque-Bera (JB)**	0.35
**Prob (Q)**	0.76	**Prob(JB)**	0.84
**Heteroskedasticity (H)**	2.63	**Skew**	0.05
**Prob (H) (two-sided)**	0.01	**Kurtosis**	3.28

Supplemental Table S5. SARIMA model fit and summary statistics for completed trials

SARIMA Summary Statistics (Completed Trials)

**Number of Observations**	48					
**AIC**	439					
**BIC**	444					
	**Coefficient**	**Standard Error**	**Z**	**P>|z|**	**[0.025**	**0.975]**
**Intercept**	152.2099	50.773	2.998	0.003	52.697	251.723
**ar.L1**	0.3951	0.180	2.189	0.029	0.041	0.749
**sigma2**	9772.1177	2227.683	4.387	0.000	5405.939	0.000

**Ljung-Box (L1) (Q)**	0.21	**Jarque-Bera (JB)**	1.45
**Prob (Q)**	0.65	**Prob(JB)**	0.49
**Heteroskedasticity (H)**	0.48	**Skew**	-0.47
**Prob (H) (two-sided)**	0.22	**Kurtosis**	3.27

Supplemental Figure S1. Derivation of the Study Database

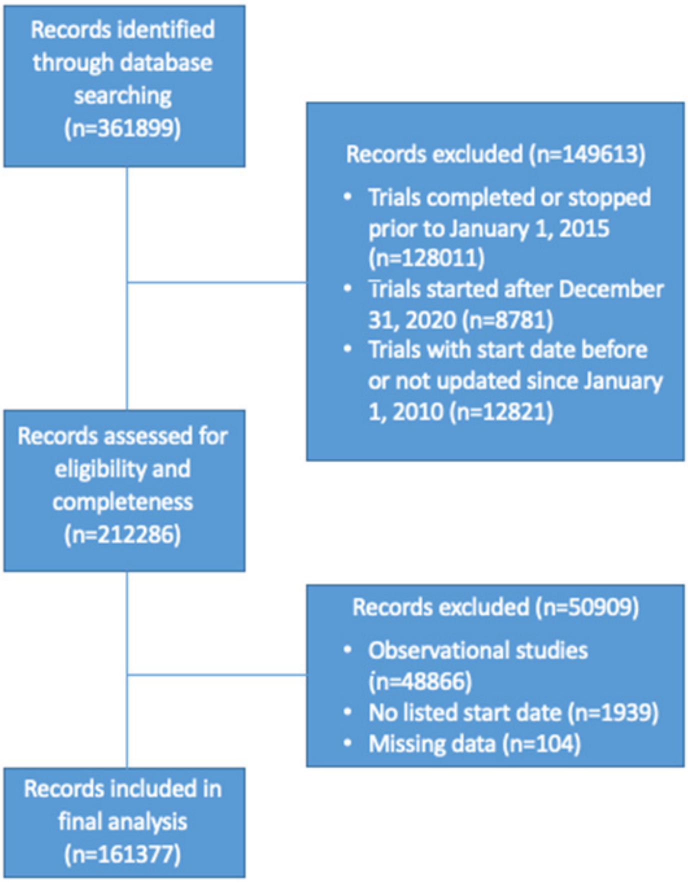

Supplemental Figure S2. Yearly number of trials initiated (blue bar), stopped (orange bar), or completed (gray bar)

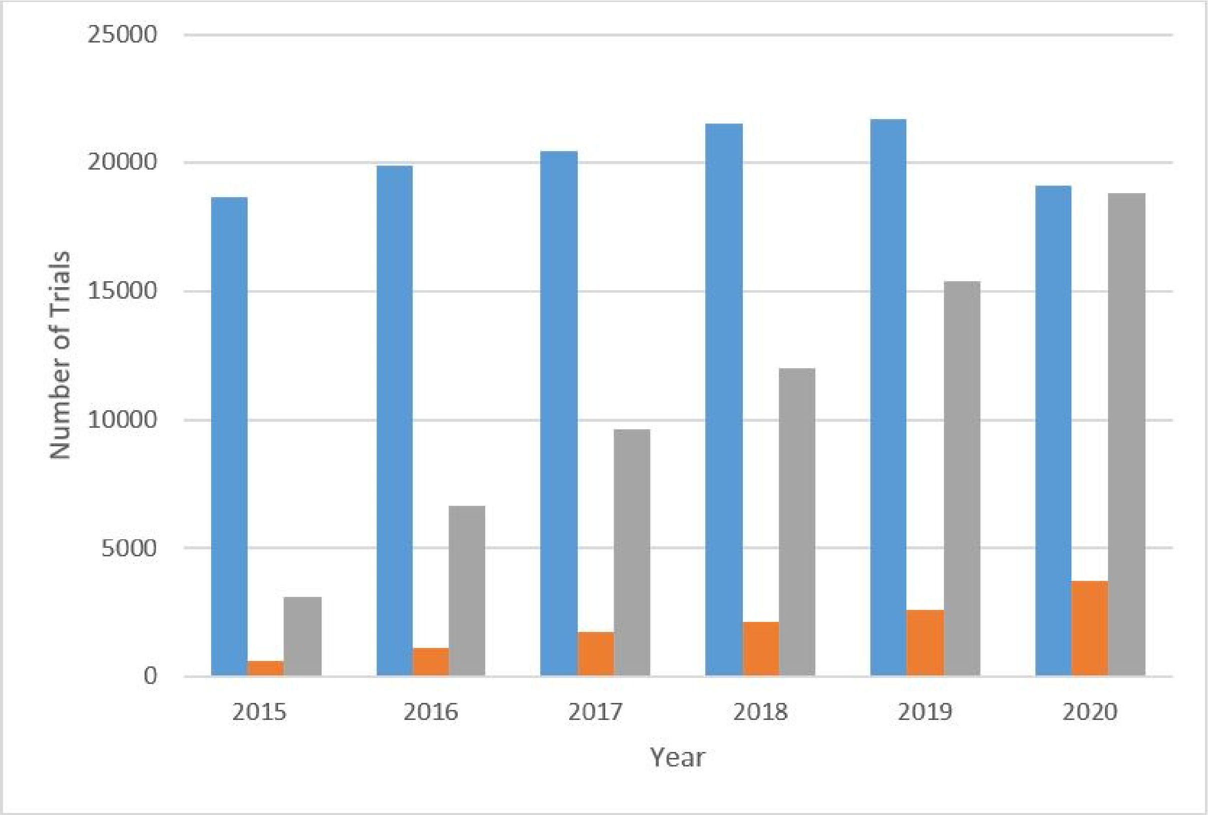

Supplemental Figure S3. Total number of trials that were active per year

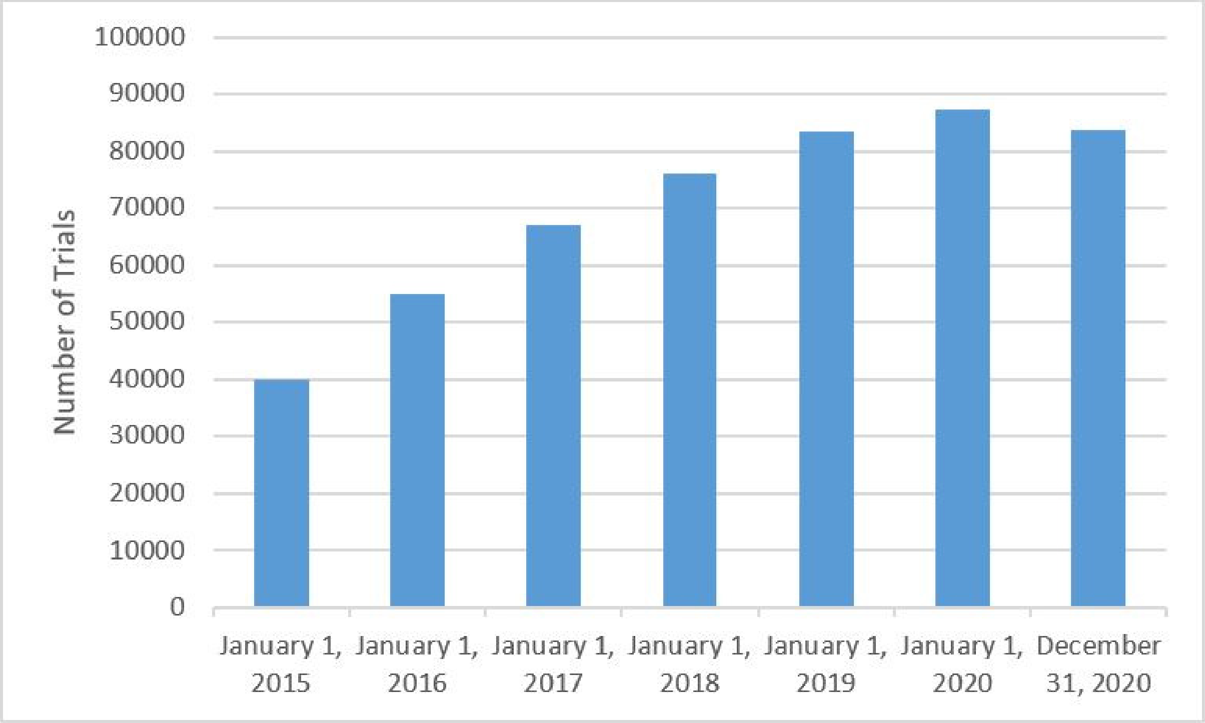

Supplemental Figure S4. (A) Total number of trials that were stopped over the study period by month. **(B)** Total number of trials that were completed over the study period by month. **(C)** Total number of trials that were initiated over the study period by month

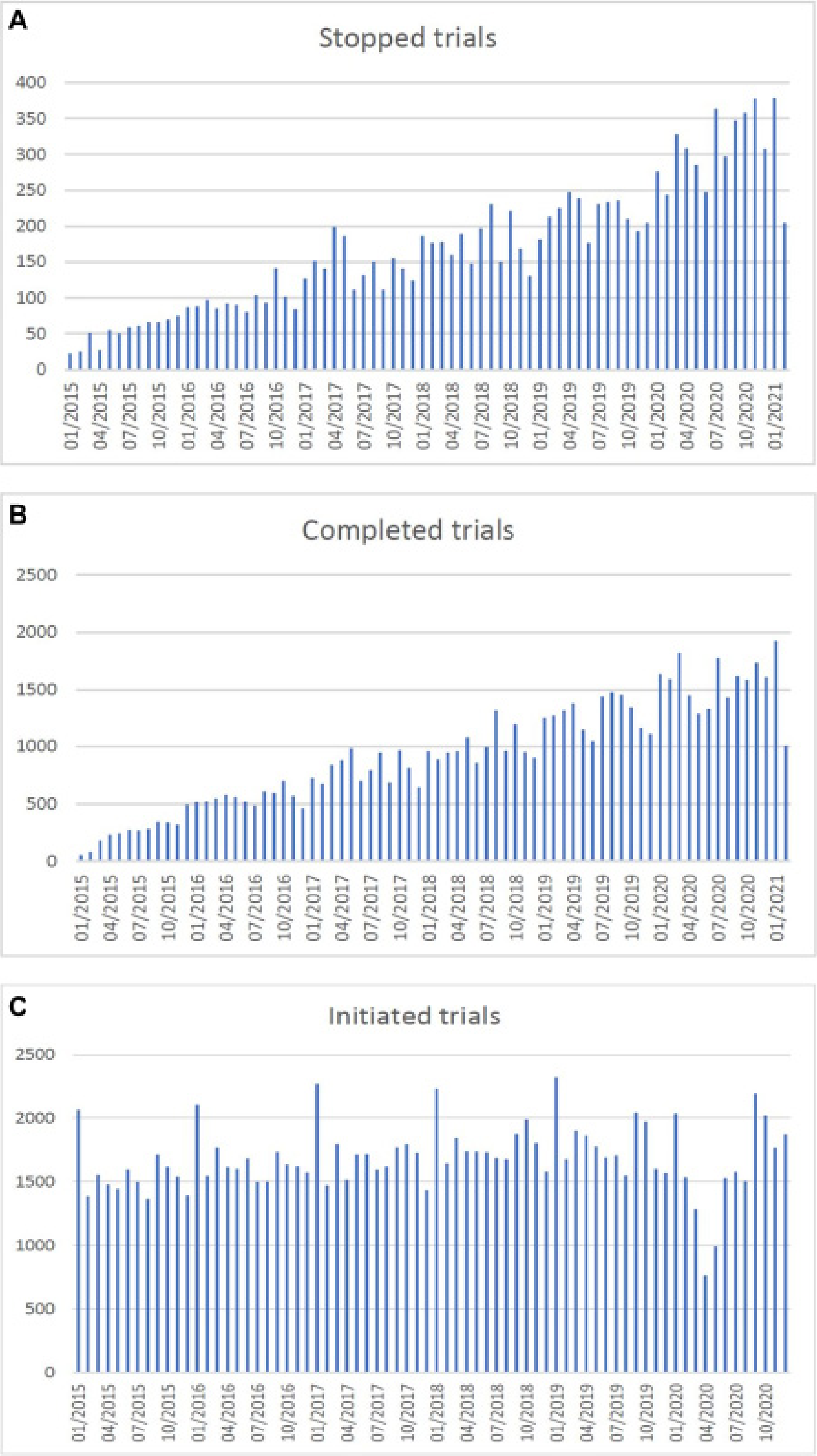

Supplement: Supplementary file 1 [file jcdd-09-00019-s001.zip › jcdd-1450720 Supplemental Material.pdf]
